# Editorial: Novel memristor-based devices and circuits for neuromorphic and AI applications, volume II

**DOI:** 10.3389/fnins.2025.1729354

**Published:** 2025-11-12

**Authors:** Heba Abunahla, Said Al-Sarawi

**Affiliations:** 1 Department of Quantum and Computer Engineering, Delft University of Technology, Delft, Netherlands; 2 School of Electrical and Mechanical Engineering, The University of Adelaide, Adelaide, SA, Australia

**Keywords:** memristor, computing, neuromorphic, energy-efficiency, artificial intelligence

This Research Topic focuses on various aspects related to the deployment of Memristor (MR) devices for neuromorphic and AI applications. Specifically, it covers the design, fabrication, and characterization of novel MR stacks that can effectively mimic the synaptic plasticity observed in neuronal behavior within the brain.

The first paper by Landsmeer et al. builds on the finding that memristive properties closely mimic the potassium and sodium ion channels in Hodgkin-Huxley-based brain models. The authors show how a volatile memristor structure made of Pt/NbOx/Ti can directly implement the Hodgkin-Huxley potassium channel. The paper starts from the memristor theoretical dynamics and demonstrates how this device reproduces the required sigmoidal gating and voltage-dependent time constants in the Hodgkin-Huxley potassium channel. This was achieved by fine-tuning the voltage and time scales of the fabricated device. The presented voltage-controlled physical memristor reports a unique implementation of the Hodgkin-Huxley potassium ion channel, providing the first direct one-to-one mapping between a device and a biological ion channel.

The second paper by Lim et al. explores a memristor-MOS transistor hardware system design that employs a memristor-transistor configuration as a 1T1R synapse. The paper reports the characteristics of a memristor when combined at the drain or source terminal of the transistor. This configuration enables seamless array expansion. The design was investigated, and corresponding design rules were proposed. Using a SPICE-based simulator, optimal transistor parameters were determined and subsequently validated through simulation. The neuromorphic learning capabilities of the proposed structure were demonstrated by implementing an SNN inference accelerator. The accuracy of the developed network was evaluated using a reduced MNIST dataset. The unique features of this structure include the use of DPI and LIF neurons, which eliminate the need for ADC and DAC modules commonly used in DNN research, resulting in improved noise performance.

The third paper by Li et al. demonstrates how analog memristors can be used in hardware event representation for event data utilization as a reservoir encoder. The authors utilized the inherent stochastic and non-linear characteristics of memristors to enable effective and low-cost feature extraction of temporal information from event streams. They also proposed a simplified memristor model and memristor-based reservoir circuit for processing dynamic visual information and feature extraction from event data. The superior accuracy of their approach was demonstrated experimentally using four event datasets, showcasing the unique application of memristor-based processing for event systems.

The fourth paper by Kipelkin et al. shows how memristor-based devices can be used to implement ion channels. They selected a simple FitzHugh-Nagumo equation system describing neuronal excitability and introduced non-linearities by the voltage-gated ion channels. Based on their approach, they generated basic excitability of a single spike, self-oscillation stable limit cycle mode with periodic spike trains, and bistability between a fixed point and a limit cycle mode. They found that due to charge accumulation effects in the memristive device, the electronic synapse qualitatively imitated real biological synapses with potentiation effects, increasing the amplitude of the response to a spike sequence.

This Research Topic provides great insight into how memristive devices can be combined with other electronic devices to better imitate or mimic biological behavior. It highlights the ability to transition from device-based to system-based approaches, where a network of these devices can be used to mimic neural networks, leading to increased use of memristor devices in neuromorphic and AI applications.

